# Noncoding RNAs are indispensable architects and regulators of biomolecular condensates

**DOI:** 10.1016/j.ncrna.2026.01.003

**Published:** 2026-01-16

**Authors:** Shiyuan Chen, Canchen Wang, Junyi Hu, Ting Luo, Qian Li, Hui Shen

**Affiliations:** School of Life Science and Technology, China Pharmaceutical University, Nanjing, 210009, Jiangsu, China

**Keywords:** Non-coding RNA, liquid-liquid phase separation, condensates, RNA-binding protein

## Abstract

Liquid-liquid phase separation (LLPS) is a biophysical mechanism by which certain biomolecules demix from the cytosol or nucleoplasm to form membraneless organelles. These droplet-like assemblies are dynamic and reversible, allowing selective enrichment of specific proteins and nucleic acids while excluding others. Classical examples include the nucleolus, P-bodies, and stress granules, all of which exhibit liquid-like behaviors such as rapid fusion, fission, and molecular exchange. Most importantly, the LLPS property has been implicated with a plethora of physiological and pathological processes. Historically, research on LLPS focused on protein drivers, especially RNA-binding proteins (RBPs) with low complexity domains or intrinsically disordered regions, contributing to multivalent weak interactions. However, it is now clear that RNA molecules especially noncoding RNAs are integral components and often active modulators of these condensates. Noncoding RNAs, including long noncoding RNAs (lncRNAs), microRNAs (miRNAs), circular RNAs (circRNAs), PIWI-interacting RNAs (piRNAs), and others, can serve as scaffolds, regulators, or clients within the LLPS droplets, thereby influencing both normal cellular organization and disease processes. This review provides an overview of current research on how ncRNAs contribute to LLPS across different cellular localizations and contexts, covering physiological condensates, disease linked phase separation, underlying molecular mechanisms, and emerging therapeutic implications.

## 1 Overview of LLPS and ncRNAs

LLPS is a biophysical process in which macromolecules demix from the surrounding cytoplasm or nucleoplasm to generate dynamic, liquid-like assemblies [1]. These condensates, including the nucleolus, stress granules, P-bodies, and nuclear speckles, enable spatial organization of biochemical reactions by concentrating specific proteins and nucleic acids while excluding others. LLPS has been recognized as a fundamental organizational principle governing diverse cellular processes, including RNA metabolism [2], DNA damage repair [3], cellular stress response [4], and immune signaling transduction [5]. Mechanistic studies have shown that LLPS is typically driven by multivalent, weak interactions among proteins and RNAs, particularly involving intrinsically disordered regions (IDR) and repetitive binding motifs [6]. In recent years, it has become clear that ncRNAs are not merely passive components of condensates but actively shape their assembly, composition, and material properties.

ncRNAs comprise diverse classes with distinct biogenesis and functions. lncRNAs are transcripts longer than ∼200 nt that often act as molecular scaffolds, recruiting multiple RBPs to nucleate or stabilize condensates [7]. miRNAs are ∼22 nt small RNAs that guide RNA-induced silencing complexes (RISCs) to target mRNAs, and their multivalent interactions can promote phase separation of silencing machinery [8]. piRNAs are slightly longer small RNAs that associate with PIWI proteins to silence transposable elements, frequently operating within specialized germline condensates [9]. circRNAs represent a unique class of covalently closed RNA molecules generated by back-splicing, which primarily rely on the cis-elements and trans-factors and lack free 5′ and 3′ ends [10,11]. This circular topology endows circRNAs with prolonged cellular persistence and the capacity to present repeated binding sites for proteins or RNAs, making them particularly well suited to modulate LLPS. Notably, several key regulators of circRNA biogenesis, such as DHX9 and FUS, possess phase-separation properties and are known to form dynamic ribonucleoprotein condensates [11,12]. This raises the intriguing possibility that there might be a regulatory feedback loop that LLPS facilitates circRNA biogenesis, while circRNAs in turn modulate LLPS properties. Together, these ncRNA species add regulatory specificity and plasticity to biomolecular condensates, enabling LLPS to function as a versatile organizing principle in both physiological and pathological contexts.

This review synthesizes recent advances in understanding how ncRNAs regulate LLPS across diverse cellular compartments and biological contexts. We discuss the roles of ncRNAs in the formation and function of physiological biomolecular condensates, examine how dysregulated ncRNA-mediated phase separation contributes to human diseases, outline the molecular and biophysical mechanisms underlying ncRNA-driven condensate assembly, and highlight emerging therapeutic strategies that target aberrant RNA-dependent phase transitions.

## 2 ncRNAs in physiological phase-separated organelles

### 2.1 Nuclear condensates: paraspeckle, Cajal body, speckle and nucleolus

**Fig. 1:**
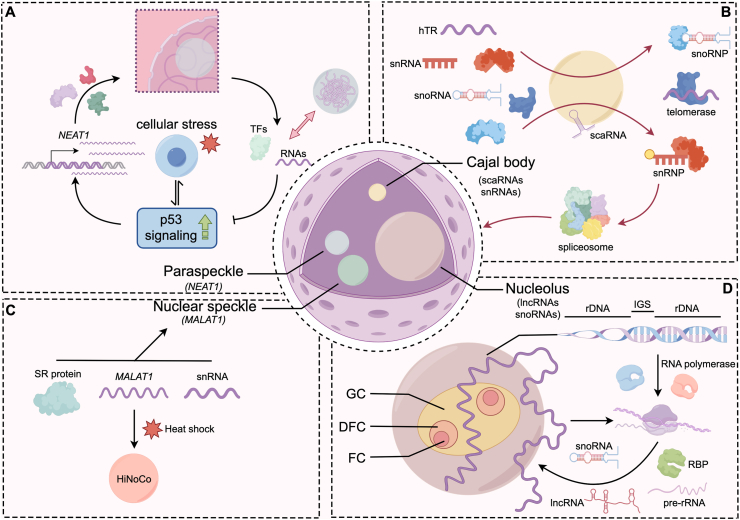
Physiological nucleolar condensates and their associated ncRNAs. (A) Paraspeckles are lncRNA-based condensates nucleated by the >20 kb scaffold RNA *NEAT1*, which recruits RBPs to form interchromatin nuclear bodies. Paraspeckles modulate gene expression by sequestering proteins and RNAs, and *NEAT1* is strongly involved in p53 signaling, replication stress responses, and chemosensitivity. (B) Cajal bodies are coilin-scaffolded nuclear condensates enriched with ncRNAs, including scaRNAs that modify snRNAs, RNAPII derived snRNAs, and transiently recruited *TERC*, collectively supporting snRNP/snoRNP biogenesis and telomerase assembly. (C) Nuclear speckles are dynamic condensates enriched with snRNAs and splicing factors. The lncRNA *MALAT1* scaffolds speckle components, including SR proteins, and maintains speckle integrity while modulating gene expression and alternative splicing. (D) The nucleolus is a tripartite LLPS-driven condensate (FC: fibrillar center; DFC: dense fibrillar component; GC: granular component) whose architecture and function rely on diverse nucleolar ncRNAs. RNAPI derived transcripts (45S pre-rRNA, snoRNAs, *PNCTR*, *IGS16*, *IGS42*) and RNAPII-derived ncRNAs (*PAPAS*, *LoNA*, *LETN*, *HOXB-AS3*, *DNAJC3-AS1*, aluRNAs, *SLERT*) collectively drive phase separation, regulate rRNA transcription and processing, and maintain nucleolar structural integrity.

Paraspeckles are a relatively newly identified subnuclear body whose assembly is driven by the lncRNA *NEAT1* [13,14]. *NEAT1* is a >20 kb RNA that acts as a scaffold for numerous RBPs [[15], [16], [17], [18]]. Paraspeckles form in the interchromatin space and harbor proteins like NONO, SFPQ, FUS, and RBM14 [13]. *NEAT1* provides multiple binding sites for these proteins, enabling their multivalent interactions and condensation into a spherical nuclear body. Indeed, *NEAT1* knockout disassembles paraspeckles, underscoring its essential scaffolding role [19]. Functionally, paraspeckles regulate gene expression by sequestering transcription factors and RNAs. Under cellular stress, such as DNA damage, p53 activation triggers *NEAT1* upregulation and paraspeckle assembly, which can modulate p53 target gene responses [20]. *NEAT1*-mediated condensates have been shown to attenuate hyperactive p53 signaling in stressed or cancer cells, affecting replication stress responses and chemosensitivity [20]. In neurobiology, stressed neurons dramatically upregulate *NEAT1*, leading to enhanced paraspeckle formation [21], and there is evidence that amyotrophic lateral sclerosis (ALS) linked mutations in the RBP FUS cause excess, abnormal paraspeckles, with *NEAT1* mislocalized, possibly contributing to neuronal dysfunction [22]. These findings highlight how a single lncRNA can nucleate a condensate with broad impacts on cell physiology and stress adaptation (Fig. 1A).

Cajal body is a membraneless nuclear organelle first observed over a century ago [23]. Cajal body is a dynamic, spherical structure and primarily participate in snRNP and snoRNP biogenesis, telomerase RNP assembly and histone mRNA 3′-end processing [[23], [24], [25]]. The signature protein of Cajal body is coilin, a scaffolding protein essential for Cajal body formation and integrity [26]. Besides, Cajal body is enriched with diverse ncRNAs, each playing a role in RNP maturation or nuclear organization. The small Cajal body-specific RNAs (scaRNAs), like U85, U87 and U93, guide site-specific modifications of small nuclear RNAs (snRNAs) before they function in the spliceosome [27]. snRNAs, like U1, U2, U4, U5 and U6, are synthesized by RNAPII and processed in Cajal body [28]. The *TERC* localizes transiently to Cajal body via its CAB box motif and TCAB1 binding, facilitating telomerase holoenzyme assembly [29] (Fig. 1B).

Nuclear speckles are dynamic nuclear bodies that contain high concentrations of splicing factors and various RNAs [30]. snRNAs and splicing factors like SR proteins are enriched in nuclear speckles to participate in the pre-mRNA splicing. Beside snRNAs, the lncRNA *MALAT1* also localizes to nuclear speckles and is required for their structural integrity [31]. *MALAT1*, ∼7 kb in length, is thought to scaffold speckle components such as SR splicing proteins, influencing gene expression and alternative splicing. Intriguingly, *MALAT1* translocates from usual speckles to form a distinct heat-inducible lncRNA-containing condensate (HiNoCo) nuclear body upon heat shock [32]. This HiNoCo is reversible and appears to protect cell viability during stress, as *MALAT1* depletion impairs cell proliferation under heat shock [32]. Thus, lncRNAs like *MALAT1* endow nuclear speckles with both stability during homeostasis and plasticity during environmental stress (Fig. 1C).

The nucleolus is the most prominent nuclear condensate and responsible for ribosomal RNA (rRNA) transcription, processing, and ribosome assembly. Recent studies have provided increasing support for the concept that the nucleolus represents a multilayered biomolecular condensate, whose formation by LLPS facilitates the initial steps of ribosome biogenesis and other functions [33]. Dysregulation of its organization is closely linked to nucleolus-associated diseases, including ribosomopathies, neurodegeneration, cancer as well as ageing [34]. Nucleolus exemplifies how abundant noncoding rRNAs themselves drive phase separation. The nascent 45S pre-rRNA transcripts, together with small nucleolar RNAs (snoRNAs) and many RBPs, phase-separate into the tripartite nucleolar sub-compartments [35,36]. Recent research has started to reveal that other long and short non-coding RNAs are not only involved in pre-rRNA transcription and rRNA production, but also shaping the organization of the multi-phase condensate [37]. The nucleolus-localized ncRNAs are derived mainly from two different transcriptional origins, RNAPI and RNAPII [38]. rDNA genes are interspersed by a 30 kb intergenic spacer (IGS) that serves as an important source of regulatory lncRNAs, such as the pyrimidine-rich noncoding transcript (*PNCTR*), *IGS16* and *IGS42*, produced by RNAPI [[39], [40], [41], [42], [43]]. It has been reported that *IGS42* lncRNA facilitates the mobility of resident proteins to contribute the elimination of nucleolus-related inclusions [43]. The biogenesis of RNAPII-derived lncRNAs in nucleolus can be categorized into two groups. One is generated locally similar to RNAPI transcripts, such as the *PAPAS*, which is transcribed by RNAPII in an antisense orientation from the IGS regions, inhibits rDNA transcription [44]. The lncRNAs in another group are transcribed from chromosome regions entirely outside of the nucleolus, including the long nucleolar ncRNA (*LoNA*) [45], *LETN* [46], *HOXB-AS3* [47], *DNAJC3-AS1* [48], aluRNAs [49], and *SLERT* (snoRNA-ended lncRNA enhances pre-rRNA transcription) [50]. Those ncRNAs all perform specific functions in the nucleolus. For example, *LoNA* has been shown to fine-tune ribosome biogenesis by coordinating rDNA transcription and rRNA methylation to meet the translational demands of long-term memory [45]. *LETN* is reported to function through NPM1-dependent regulation of nucleolar structure, supporting proliferation by maintaining nucleolar homeostasis [46]. In addition, *DNAJC3-AS1* is reported to retain the prion-like proteins FBL proteostasis and maintain the state of FC/DFC units [48]. (Fig. 1D).

In summary, nuclear condensates such as the nucleolus, speckles, paraspeckles, and Cajal bodies consistently rely on ncRNAs as architectural scaffolds, highlighting ncRNA's conserved role as both a structural and regulatory determinant of nuclear organization and function.

### 2.2 Cytoplasmic condensates: stress granule, p-body, and germ granule

**Fig. 2:**
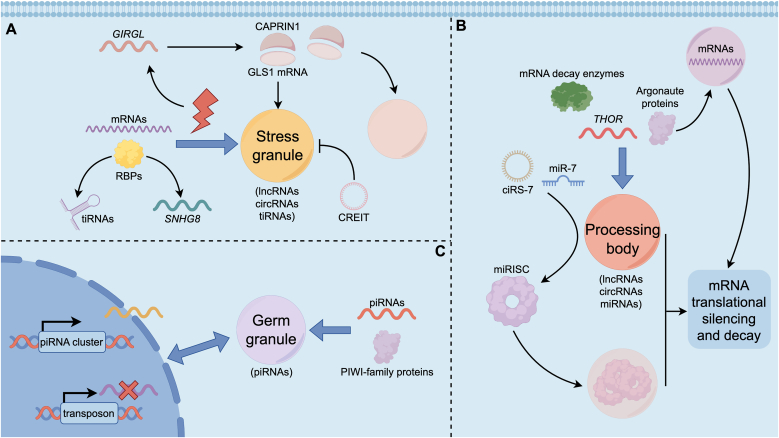
Physiological condensates in cytoplasm and their associated ncRNAs. (A) In the cytoplasm, stress induces untranslated mRNAs and RBPs to form SGs. Also, lncRNA *GIRGL* can be induced to integrate a complex between dimers of CAPRIN1 and *GLS1* mRNA, promoting LLPS of CAPRIN1 and SG assembly. CircRNA *CREIT* exhibit suppression effect on SG assembly. LncRNA *SNHG8* and tiRNAs can bind and compete RBPs from SG. (B) P-bodies concentrate mRNA decay enzymes, lncRNAs such as *THOR*, AGO proteins, and miRNAs. The circRNA *ciRS-7* recruits *miR-7* to enhance RISC clustering, while AGO proteins can phase-separate to form droplets that sequester target mRNAs. These condensates collectively promote mRNA silencing and decay. (C) PIWI-family proteins and piRNAs can form germ granule which regulates transposon silencing and the onset of piRNAs from single strand transcript.

In the cytoplasm, ncRNAs also play key roles in forming RNP granules during various conditions. Stress granules (SGs) are transient cytoplasmic droplets that form under stress, such as heat, oxidative stress and viral infection, when translation is globally repressed [51]. SGs consist of untranslated mRNAs, stalled initiation complexes, and RBPs like G3BP1, TIA1, and YBX1 [[52], [53], [54]]. While mRNAs are the core scaffolds of SGs, ncRNAs can also help nucleate, stabilize and modulate SG behaviors [55]. In tauopathies, lncRNA *SNHG8* can bind TIA1 to compete TIA1 from SG, leading to less SG assembly [56]. The lncRNA *GIRGL* (glutamine insufficiency regulator of glutaminase lncRNA), induced upon glutamine starvation, drives formation of a complex between dimers of CAPRIN1 and GLS1 mRNA, serving to promote LLPS of CAPRIN1 and inducing SG formation [57]. Besides, it has been reported that circRNA *CREIT* exhibit suppression effect on SG assembly, which overcomes doxorubicin resistance in triple-negative breast cancer by destabilizing PKR [58]. Moreover, certain small ncRNAs can suppress SG formation. For instance, tRNA-derived fragments (tiRNAs) have been shown to bind YBX1 and prevent it from nucleating SGs, thereby tuning the stress responses [59] (Fig. 2A).

Processing body (P-body) is another type of cytoplasmic condensate, enriched for mRNA decay enzymes, such as DCP1/DCP2 decapping complex and XRN1 exonuclease, and the microRNA-induced silencing machinery [60]. P-body forms constitutively in many cells and become more pronounced during stress or translational repression. Large scale purification of P-body has allowed profiling of their RNA complement, revealing that beyond mRNAs, P-body is also enriched in ncRNAs [61]. Single-molecule tracking studies have shown that lncRNAs, such as *THOR*, can localize to P-body, yet the functional implications of this localization need further investigation [62]. P-body is also a hub where microRNA loaded RISCs congregate to mediate mRNA translational silencing and decay [62]. The Argonaute proteins (AGO1-4 in humans) harbor low complexity regions and glycine rich motifs that promote condensation. Remarkably, it was demonstrated that AGO2 alone can undergo LLPS in vitro and in cells, forming droplets that sequester target mRNAs [8]. This suggests RISC has an intrinsic propensity to phase separate when bound to multiple target sites. A recent study in human cells shows that *ciRS-7*, a circular RNA containing multiple binding sites for miR-7, can enhance such RISC clustering, thereby increasing multivalent interactions among RISCs and driving their condensation [63] (Fig. 2B).

Specialized germ cell granule represents another arena where ncRNAs and LLPS are intimately connected [64]. In *C. elegans* germ cells, germ granule, also called P-granule, is perinuclear condensate required for germline development and transposon silencing. Germ granule consists of PIWI-family proteins, such as PRG-1, piRNAs, and other small RNA pathway components. piRNAs are a cluster of small ncRNAs derived from single strand transcript, regulating the silence of mobile elements and guarding germline maintenance. In germ cells, piRNAs and piRNA biogenic factors are enriched in germ granules for the onset of piRNA, which is involved in the genome integrity [65]. Linking to P-body, piRNA factors would be separated into germ granule and P-body, and the adequate demixing of two above can prevent piRNAs from aberrant overexpression based on non-transposable elements, demonstrating the importance of proper compartmentalization on germ granule and P-body for safeguarding piRNA biogenesis and effective functions [66]. Some researchers also discover a specific protein in P-body engage in germ granule condensation and piRNA ping-pong circle [67] (Fig. 2C).

In summary, diverse cytoplasmic condensates rely on ncRNAs not only as passive cargos but as active regulators that scaffold, modulate, and coordinate RNA-protein phase separation processes, thereby finetuning cellular stress responses, mRNA turnover, and germline genome integrity.

## 3 Molecular mechanisms: how ncRNAs modulate phase separation

**Fig. 3:**
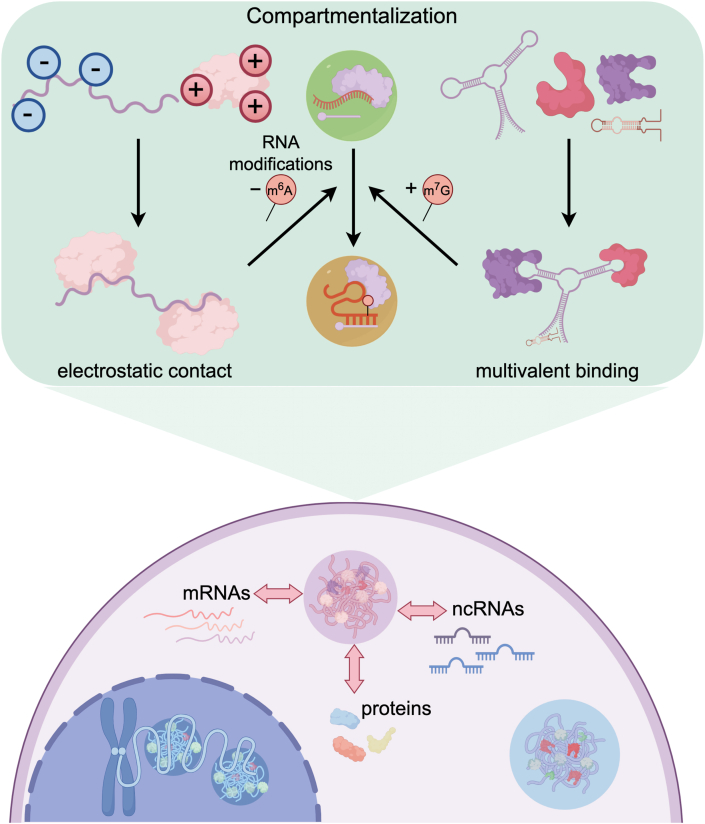
Molecular mechanisms underlying how ncRNAs modulate phase separation. ncRNAs contribute to LLPS through several molecular mechanisms that provide the necessary valency and specificity for condensate formation. One ncRNA molecule can bind several RBPs simultaneously via electrostatic contacts and sequence specific motifs. Stem-loop motif as a pervasive RNA secondary structure, contributes to phase-separating RBPs through a multivalent binding mode. Besides, RNA modifications, such as m^6^A demethylation and m^7^G methylation, promote the assembly of condensates through complex RNA-protein and RNA-RNA interaction.

ncRNAs contribute to LLPS through several molecular mechanisms that provide the necessary valency and specificity for condensate formation (Fig. 3).

### 3.1 Multivalent binding and scaffolding

RNAs can serve as scaffolds that bring together multiple proteins. The polyanionic RNA backbone enables one RNA molecule to bind several RBPs simultaneously via electrostatic contacts and sequence specific motifs. lncRNAs in particular have a large footprint for binding. For example, the cytoplasmic lncRNA *NORAD* contains multiple Pumilio response elements, allowing it to bind several PUM1/PUM2 proteins and sequesters them in the condensate [68]. Similarly, *HSATIII* repeat lncRNA transcripts provide a binding platform for many HNRNPA1 proteins, which contain low-complexity prion-like domains and can form condensates to nucleate nuclear stress bodies, compartmentalizing splicing and heat-shock protein mRNAs [69]. The X-inactive specific transcript (*XIST*) serves as anther classic example that exerts its biological functions by acting as a molecular scaffold to mediate LLPS [70]. *XIST* recruits silencing molecules, most of which contain RNA-binding domains or IDRs, and facilitates their binding to specific conserved repetitive elements within its own RNA. This process enables the accumulation of repressive histone marks, specifically trimethylation of histone H3 on lysine 27 (H3K27me3) [71], on the inactive X chromosome. Subsequent studies have demonstrated that the RBPs PTBP1, MATR3, TDP-43 and CELF1 are capable of interacting with the E-repeat sequences of *XIST* through a multivalent binding mode, thereby facilitating the formation of phase-separated droplets. These droplet structures induce the generation of functional compartments within the cell nucleus, which in turn drives the silencing of genes associated with X-chromosome inactivation [72,73]. In these cases, the lncRNA acts as an architectural anchor, without which the condensate cannot form. Likewise, circRNAs can scaffold proteins and function in diverse biological processes [74,75]. *circSPECC1*, derived from SPECC1, recently is reported to bind the autophagy enzyme ATG4B at two sites and simultaneously bind its E3 ligase RNF5, thereby scaffolding a ubiquitination reaction within a condensate [76]. Through this mechanism, *circSPECC1* promotes ATG4B LLPS and enhances ATG4B ubiquitination and turnover. The *MajSAT* has also been demonstrated to function as a molecular scaffold, which, together with SAFB, organizes the formation of biomolecular condensates through complex RNA-protein and protein-protein interaction networks, which promotes phase separation and thus maintains the high-level structure of heterochromatin [77]. Overall, RNAs function as one long molecule that can tether many clients, dramatically raising local concentrations and favoring phase transition once a threshold is reached (Fig. 3).

### 3.2 Secondary structure

ncRNAs are not just linear scaffolds, their ability to fold into secondary or tertiary structures adds another layer of multivalency. G-quadruplexes (G4), hairpin loops, and double-stranded RNA can all serve as interaction surfaces for proteins or other RNAs. For instance, G4 structures in RNAs often attract RGG/RG-rich domains of RBPs [78]. The notorious GGGGCC repeat RNA in the C9orf72 gene, implicated in amyotrophic lateral sclerosis/frontotemporal dementia (ALS/FTD), forms G4 that promotes phase separation of various RBP in vitro and in cells [[79], [80], [81]]. These repeat RNAs form nuclear foci that aberrantly sequester splicing factors and other RBPs, functioning like pathological condensates. Besides, stem-loop motif is a pervasive RNA secondary structures, known to be recognized by phase-separating RBPs and play a critical role in the recruitment and condensation of RNA into liquid-like droplets [82]. The lncRNA *SLERT* is a unique type of long ncRNA that is flanked by box H/ACA snoRNAs at both ends plus three internal loops. Among these structural elements, loop 3 serves as the key functional domain for its binding to the DEAD-box RNA helicase DDX21. Notably, DDX21 can only interact effectively with structured *SLERT*, whereas denatured *SLERT* loses this binding capability. Through this specific secondary structure, *SLERT* induces the conformational transition of DDX21 from an open state to a closed state via a molecular chaperone-like mechanism, reducing DDX21 multimerization and promoting the formation of loose clusters. In turn, this regulatory process modulates the phase separation properties of nucleolar FC/DFC units [83]. Meanwhile, studies by John A. Tainer and colleagues have demonstrated that the secondary structure of lncRNA *LINP1*, comprising three stem-loops and one G4, serve as the core regulatory basis for its involvement in phase separation [84]. Specifically, G4 drives *LINP1* to form 10–24 nm phase-separated condensates in vitro, while the stem-loop structures assist in maintaining RNA flexibility to promote the dynamics of assembly. Additionally, DNA damage can induce intracellular *LINP1* to form large aggregates through RNA-RNA interactions, providing a locally concentrated environment for the non-homologous end joining repair factors (Fig. 3).

### 3.3 RNA modifications

Post-transcriptional modifications of RNA can regulate LLPS propensity as well. m^6^A is a common modification on mRNAs and lncRNAs that influences structure and protein binding. Modified RNAs can recruit specific readers that participates in phase separation. A striking example is enhancer RNAs (eRNAs) transcribed at super-enhancers. These eRNAs are heavily m^6^A modified, which enables binding of the m^6^A reader YTHDC1 [[85], [86], [87]]. YTHDC1 in turn forms a condensate at the enhancer that helps concentrate co-activators like BRD4, thereby boosting transcription of nearby genes [85,86]. Knocking down the eRNA or the m^6^A writers disrupts YTHDC1 focus formation and reduces target gene expression. Besides, m^6^A modification status of lncRNA *NEAT1* serves as a key molecular switch that regulates the phase separation properties of paraspeckles [88]. Relevant studies have demonstrated that under hypoxic stimulation, the m^6^A demethylase ALKBH5 rapidly accumulates in the paraspeckle region and prioritizes the initiation of m^6^A demethylation of *NEAT1*. This demethylation process enhances the stability of *NEAT1*, thereby promoting the assembly of paraspeckle under hypoxic conditions and significantly improving the efficiency of phase separation [89]. In cytoplasm, studies by Catherine Rabouille and colleagues revealed that ncRNAs in SGs tend to form higher-order aggregates. They hypothesized that if ncRNAs undergo modifications such as m^7^G, their RNA-RNA interactions may be enhanced, thereby promoting the formation and stabilization of SG condensates [90]. Overall, the epitranscriptomic state modulates the stickiness and conformation of ncRNAs, influencing how and when they engage in LLPS (Fig. 3).

In summary, ncRNAs are key organizers and regulators of LLPS. Their negative-charge backbones and flexible structures confer multivalency, their structured motifs and repetitive elements provide binding platforms, and their dynamic modification allows condensates to form only under the right conditions.

## 4 Phase separation of ncRNAs in disease contexts

### 4.1 Oncogenic and tumor-suppressive condensates in cancers

**Fig. 4:**
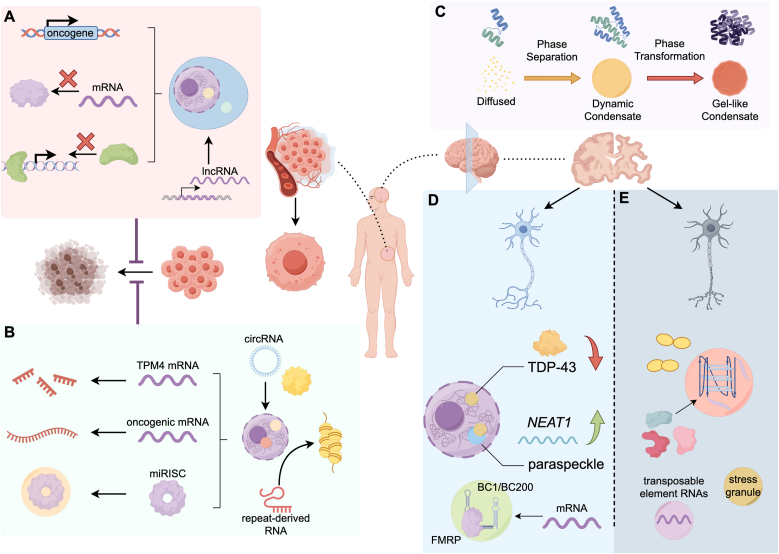
Phase separation of ncRNAs in disease contexts. (A) In cancer cells, several lncRNAs can be amplified and overexpressed, then bind and induce phase separation in nucleus and cytoplasm, thereby activating the oncogenes, suppressing the translation of genes that hinder cancer cells development, and limiting damage-related signaling. (B) The *circASH2* binds to YBX1 in the nucleus and enhances phase separation, which leads to more efficient decay of TPM4 mRNA. The *circRNF13* and *circCLASP2* enhance phase separation of IGF2BP1 and DHX9 respectively, stabilizing oncogenic mRNAs via LLPS. The repeat-derived RNAs can nucleate phase-separated foci at perinucleolar regions, resulting in a cancer-specific heterochromatin body. All those mechanisms can restrain cancer cells undergoing apoptosis under stress. (C) In neurodegenerative diseases, some pathological RBPs can undergo LLPS and convert to irreversible aggregates with ncRNAs involved. (D) In normal neurons, *NEAT1* upregulation correlates with fewer cytoplasmic TDP-43 aggregates, which ameliorates TDP-43 toxicity. The lncRNA *BC1/BC200* interacts with FMRP, potentially forming small translational control granules to prevent synaptic dysfunction. (E) In pathological neurons, transposable element RNAs are aberrantly expressed. *miR-128* regulate TIA1, affecting SG assembly in AD models. The intronic GGGGCC repeats produce toxic RNA foci in the nucleus, sequestering numerous RBPs through G4, thus compounding C9orf72 linked neurodegenerative disorders.

Cancer cells commonly hijack phase separation processes to reprogram gene expression and signaling. Numerous oncogenic ncRNAs have been found to drive condensate formation that supports tumor progression [91,92]. A striking recent example is the pseudogene-derived lncRNA *ZNF252P*, which is amplified and overexpressed in many solid tumors [93]. *ZNF252P* was shown to bind and induce phase separation of HNRNPK and ILF3 in the nucleus and cytoplasm, respectively. Through this dual condensate mechanism, *ZNF252P* activates the oncogene *c-Myc* at multiple levels [93]. Another notable lncRNA is *NEAT1*, introduced earlier for paraspeckles. *NEAT1* is frequently upregulated in cancers and its paraspeckle-forming ability has been linked to tumor cell survival under stress [94]. In a landmark study, p53-induced *NEAT1* was found to help cancer cells survive replication stress by modulating p53 activity [20]. High *NEAT1* levels in tumors correlate with therapy resistance, possibly because *NEAT1* condensates sequester pro-apoptotic factors or limit DNA damage signaling [95]. LncRNA *MALAT1* is also associated with the dysregulation of phase separation process in cancer. It has showed that *MALAT1*'s heat-induced condensates support cell survival under acute stress, which could help tumor cells endure fever or inflammation in the tumor microenvironment [32]. lncRNAs can directly regulate signaling by nucleating phase-separated complexes as well. *SNHG9*, a phosphatidic acid binding lncRNA, can bind the signaling lipid PA and induce LLPS of the Hippo pathway kinase LATS1. These puncta prevent LATS1 from phosphorylating and inhibiting YAP, thereby enhancing pro-tumor YAP oncogenic signaling [96]. Another lncRNA, *GIRGL*, is induced under nutrient stress and helps cancer cells adapt metabolically. Under glutamine starvation, *GIRGL* binds and dimerizes CAPRIN1 with GLS1 mRNA to form condensates, which suppresses translation of GLS1, slowing glutamine metabolism and allowing cancer cell survival during nutrient deprivation [57] (Fig. 4A).

Circular RNAs can also function as oncogenes or tumor suppressors via LLPS. The circRNA *circASH2*, derived from the ASH2L gene, was recently identified as a metastasis suppressor in hepatocellular carcinoma. Mechanistically, *circASH2* binds to YBX1 in the nucleus and dramatically enhances its phase separation, which leads to more efficient decay of *TPM4* mRNA, encoding an actin binding protein that facilitates cytoskeletal organization and tumor cell invasion [97]. Additionally, *circVAMP3* has been identified as a tumor-suppressive circRNA that inhibits hepatocellular carcinoma (HCC) progression through modulation of phase separation as well. *circVAMP3* is downregulated in HCC and exerts its anti-tumor function by promoting CAPRIN1-dependent phase separation and stress granule assembly, where *circVAMP3*, CAPRIN1, and c-Myc co-localize, resulting in translational repression of c-Myc under stress [98]. Conversely, many circRNAs act as oncogenes via LLPS as well. The *circRNF13* and *circCLASP2* have been reported to enhance phase separation of IGF2BP1 and DHX9 respectively, stabilizing oncogenic mRNAs in oral cancer or nasopharyngeal carcinoma [99,100]. Another high profile circRNA *ciRS-7*, overexpressed in some cancers, can cluster RISC into condensates, which might influence DNA repair and radio resistance, potentially giving cancer cells a survival advantage after radiation therapy [63]. Thus, circRNAs can contribute to post-transcriptional gene regulation and DNA damage response in cancer cells via LLPS (Fig. 4B).

Beyond aforementioned ncRNAs, repeat-encoded noncoding transcripts also contribute to LLPS phenomena in cancer. Transcripts from the *HSATII* pericentromeric repeats are abnormally expressed in some cancers and accumulate in the nucleus. These repetitive RNAs nucleate phase-separated foci at perinucleolar regions, which selectively sequester the polycomb repressive complex 1 (*PRC1*) and other chromatin proteins, which results in a cancer-specific heterochromatin body, disrupting normal epigenetic regulation [101].

### 4.2 Neurodegenerative and neurological disorders

Many neurodegenerative diseases are now understood to involve pathological phase transitions of RBP, often in association with ncRNAs (Fig. 4C). ALS is closely linked with TDP-43, which can undergo LLPS in disease and convert to irreversible aggregates. Some studies suggest that lncRNA *NEAT1* plays a protective role by sequestering toxic proteins. TDP-43 forms aberrant nuclear foci in stressed neurons that partially colocalize with paraspeckles, and *NEAT1* upregulation correlates with fewer cytoplasmic TDP-43 aggregates [102,103]. Overexpression of the short *NEAT1_1* isoform has been reported to ameliorate TDP-43 toxicity in cellular models [104]. These findings are still emerging, but they hint that modulating lncRNA-mediated condensates could alter neurodegenerative progression (Fig. 4D).

Another major contribution of ncRNAs in neurodegeneration comes from repeat expansion disorders. In *C9orf72* linked neurodegenerative disorders, the intronic GGGGCC repeats produce toxic RNA foci in the nucleus. These RNA foci have been shown to sequester numerous RBPs through G4 [79,105,106]. Moreover, the dipeptide repeat (DPR) proteins translated from the *C9orf72* repeat can alter phase separation of RBPs, compounding the problem [107,108]. These condensates likely disrupt normal nuclear organization, leading to widespread gene expression changes in neurons. Targeting the sense and antisense *C9orf72* repeat RNAs with antisense oligonucleotides has been shown to reduce or clear these foci [109], which is a promising therapeutic strategy currently in clinical trials (Fig. 4E).

Beyond ALS, Alzheimer's disease (AD) and other tauopathies involve granules as well. *miR-128* has been reported to regulate an RBP TIA1, affecting SG assembly in AD models [110]. Transposable element RNAs, usually kept suppressed by piRNAs and other pathways, are aberrantly expressed in some neurodegenerative diseases, and these RNAs might engage LLPS pathways or trigger innate immune responses via condensates [111]. Importantly, not all condensates in neurons are harmful. Some are physiological and essential with ncRNAs found there too. The brain-specific lncRNA BC1/BC200 localizes to synapto-dendritic regions and interacts with FMRP, potentially forming small translational control granules. Loss of such ncRNA-containing granules might contribute to synaptic dysfunction in diseases like fragile X syndrome or AD [112].

Collectively, the nervous system exemplifies a delicate equilibrium where ncRNA-driven condensates must be tightly regulated. Perturbations can push these assemblies from liquid to gel/solid states, leading to neurodegenerative diseases. Unraveling the ncRNA components in these assemblies is an active area, with the hope that stabilizing beneficial condensates or dissolving toxic ones could be a new therapeutic angle for neurodegenerative diseases.

## 5 Therapeutic applications

Given the significant roles of ncRNAs in LLPS, which has been implicated in various diseases, researchers are actively exploring ways to modulate these condensates by targeting ncRNAs for clinical benefits. Antisense oligonucleotides (ASOs) can directly target pathogenic ncRNAs that drive harmful phase separation. For instance, ASOs against the *C9orf72* repeat RNAs have shown efficacy in reducing toxic RNA foci and DPR proteins in ALS models [113]. Similarly, ASOs against *MALAT1* were advanced to clinical trials for lung cancer, aiming to blunt metastasis by disassembling *MALAT1* speckles [114]. Additionally, small molecules that can interfere with critical RNA-protein interactions or modify the biophysical environment of condensates are of importance. One intriguing example is Lopinavir, that has been found to increase *circSPECC1* level and consequently restores the scaffold needed to induce ATG4B LLPS and degradation, thereby suppressing autophagy-mediated metastasis in a preclinical model [76]. This suggests that we might screen drug libraries for those that alter expression or stability of beneficial ncRNAs. Furthermore, introducing engineered RNAs that can compete with or replace pathological ones is a promising idea to treat dysregulated condensation. For example, delivering RNA oligonucleotides that can bind FUS and TDP-43 proteins exhibited reversed FUS and TDP condensation and fibrillization in vitro and in cells. Since short RNAs can be effectively delivered to the human brain, these oligonucleotides could have therapeutic utility for ALS/FTD and related disorders [115]. These interventions showcase how LLPS mediated diseases can be treated at the RNA level by knocking down the scaffold RNA and effectively melting the condensate.

Taken together, as our understanding deepens, ncRNAs and their phase-separation behavior are becoming part of the clinical vocabulary, bridging molecular cell biology and translational medicine.

## 6 Conclusion and future perspectives

In the past few years, we have gained a profound appreciation for the roles of ncRNAs in organizing the spatiotemporal landscape of the cell via LLPS. ncRNAs operate as the molecular glue and scaffolding in many condensates, from the nucleus to the cytoplasm. They endow condensates with specificity through sequence motifs and structures and responsiveness through regulated expression, making phase separation a tunable cellular process. This has prominent implications that many cellular decisions, such as gene expression programs, stress responses, and developmental transitions, are governed by certain condensates, and ncRNAs are important players in those processes.

ncRNAs not only sculpt physiological condensates but, when dysregulated in expression, sequence, structure, or post-transcriptional modification, can drive the formation of aberrant, dysfunctional phase-separated assemblies that compromise cellular homeostasis. In disease states, such ncRNA-mediated condensates may sequester essential RBPs, titrate away regulatory RNAs, or alter local translation, thereby rewiring core pathways involved in gene expression, chromatin organization, and stress signaling. This pathological condensation is increasingly recognized as a unifying mechanism across a wide spectrum of disorders.

In conclusion, ncRNAs are indispensable architects and regulators of biomolecular condensates, influencing health and disease across multiple organisms. There will likely be even more types of ncRNAs awaiting discovery that contribute to LLPS, as well as more diseases in which dysregulated condensates play roles. Interdisciplinary efforts that integrate high resolution cell biology with genomics and biophysics will not only deepen our fundamental understanding of cell organization but also pave the way to innovative treatments by targeting the RNA molecules at their core. Such interventions could transform how we approach complex diseases, making the study of ncRNAs and LLPS one of the most thrilling frontiers in molecular medicine.

## CRediT authorship contribution statement

**Shiyuan Chen:** Writing – original draft. **Canchen Wang:** Writing – original draft. **Junyi Hu:** Writing – review & editing. **Ting Luo:** Supervision. **Qian Li:** Writing – review & editing, Supervision. **Hui Shen:** Project administration, Conceptualization.

## Declaration of competing interest

The authors declare that they have no known competing financial interests or personal relationships that could have appeared to influence the work reported in this paper.
